# Comparative performance of Kato–Katz, POC-CCA and real-time PCR in detecting *Schistosoma mansoni* infection at different endemicity settings in northwest Ethiopia: a cross-sectional study

**DOI:** 10.1186/s41182-025-00777-7

**Published:** 2025-08-04

**Authors:** Getaneh Alemu, Endalkachew Nibret, Abaineh Munshea, Melaku Anegagrie, María Flores-Chávez, Tadesse Hailu, Arancha Amor

**Affiliations:** 1 Department of Medical Laboratory Science, College of Medicine and Health Sciences, Bahir Dar University, PO.BOX: 79, Bahir Dar, Ethiopia; 2 Biology Department, Science College, Bahir Dar University, Bahir Dar, Ethiopia; 3 Health Biotechnology Division, Institute of Biotechnology (IoB), Bahir Dar University, Bahir Dar, Ethiopia; 4 Mundo Sano Foundation and Institute of Health Carlos III, Madrid, Spain; 5 Reference and Research Laboratory in Parasitology. National Center for Microbiology, Instituto de Salud Carlos III, Madrid, Spain

**Keywords:** *Schistosoma mansoni*, Performance, KK, POC-CCA, RT- PCR, Ethiopia

## Abstract

**Background:**

Effective detection of *Schistosoma mansoni* is crucial for the control and management of the infection. This study aimed to evaluate the field performance of three diagnostic methods—point-of-care circulating cathodic antigen (POC-CCA) assay, Kato–Katz (KK) technique, and real-time polymerase chain reaction (RT-PCR)—for detecting *S. mansoni* infection in different transmission settings across northwest Ethiopia.

**Methods:**

From February to June 2023, a cross-sectional study was conducted in the Amhara Regional State of Ethiopia, involving 1192 randomly selected participants. Stool samples were analyzed using the KK technique and RT-PCR, while urine samples were tested using the POC-CCA cassette. The performance of POC-CCA and RT-PCR was evaluated against a KK reference standard across varying transmission areas. In addition, diagnostic accuracy for all three methods was assessed using latent class analysis (LCA) with Mplus software. Sensitivity, specificity, positive predictive value (PPV), negative predictive value (NPV), and kappa statistics were calculated using the Simple Interactive Statistical Analysis (SISA) online tool.

**Results:**

The KK method showed the lowest prevalence (33.4%) and demonstrated reduced sensitivity, particularly in low (54.6%) and moderate (67.0%) transmission areas, though it performed better (88.6%) in high-endemic settings compared to LCA reference. In contrast, the POC-CCA test showed higher prevalence (53.5%) and consistently high sensitivity (93.4–100%) across transmission settings, although its specificity declined in low (86.0%) and moderate (78.9%) endemic areas against LCA. Compared to the KK gold standard, POC-CCA had high sensitivity (93.5%) and NPV (95.3%) but lower specificity (62.5%) and moderate agreement (kappa = 0.52). RT-PCR exhibited strong diagnostic performance, with high sensitivity against both KK (93.5%) and LCA (97.2%) but declining specificity as endemicity increased (84.2% in low, 79.4% in moderate and 28.0% in high-endemic areas), and showed substantial agreement with LCA (kappa = 0.75).

**Conclusions:**

The KK demonstrates low sensitivity, particularly in low-transmission settings. Both the POC-CCA test and RT-PCR show good performance for detecting *S. mansoni* infection. However, due to its complexity and resource requirements, RT-PCR is not feasible for routine field use. Therefore, we recommend the adoption of the POC-CCA test in Ethiopia’s SCH control and elimination programs.

**Supplementary Information:**

The online version contains supplementary material available at 10.1186/s41182-025-00777-7.

## Background

Schistosomiasis (SCH), caused by the trematode worm *Schistosoma* species, remains a major scourge for humankind. Every year, the parasite infects approximately 250 million people in low- and middle-income countries [1, 2] mainly affecting sub-Saharan Africa, the Middle East, Asia and Latin America [3]. In Ethiopia, among the 966 districts mapped between 2013 and 2020, 480 were endemic, with 229 low-endemic (1–9.9% prevalence), 173 moderate-endemic (10–49.9%), and 78 high-endemic (≥ 50%) districts. Although both *S. mansoni* and *S. haematobium* are present, *S. mansoni* is more widely distributed throughout the country [4].

To address this, Ethiopia has adopted the World Health Organization’s (WHO) 2021–2030 road map for the control and elimination of SCH [5]. The Federal Ministry of Health has been implementing a multi-pronged approach to control SCH, including mass drug administration, facility-based case diagnosis and treatment, snail control, water, sanitation, and hygiene initiatives, and social and behavioral change communications.

Accurate diagnostic tests are crucial for mapping, case diagnosis, and monitoring the effectiveness of control programs. While several methods are used to detect *S. mansoni*, the Kato–Katz (KK) technique is considered the gold standard, and it is widely used in endemic countries, including Ethiopia [4–6]. However, KK has low sensitivity, especially in low-transmission areas and low-intensity infections [7–9]. Its performance is also affected by daily fluctuations in egg excretion, single-sex and pre-patent infections [10–12]. Therefore, as countries shift from control to elimination efforts, a more precise point-of-care test capable of detecting light and early infections is needed.

The point-of-care circulating cathodic antigen (POC-CCA) test is a promising diagnostic tool [13]. The circulating cathodic antigen (CCA) is produced by live juvenile and adult *Schistosoma* worms and excreted in urine, clearing within three weeks after treatment. As such, detecting CCA indicates the presence of live worms and active infection. Following the WHO guidance, POC-CCA has been used alongside KK for mapping and monitoring control programs in some countries [6, 14]. Studies in *S. mansoni* endemic areas have shown that POC-CCA has superior sensitivity compared to routine KK [15–17]. However, further research indicates that POC-CCA may not be as sensitive in low-transmission settings [18], highlighting the need for more data before it can be recommended for widespread use. The POC-CCA test is not routinely used in Ethiopia, and its performance is not well addressed to support future use.

Polymerase chain reaction (PCR) is considered more accurate than both KK and POC-CCA for detecting schistosomes [19, 20], though some studies have reported variable specificity [21, 22]. Due to its high cost, complex laboratory requirements, and need for skilled personnel, PCR is typically used only in research and for evaluating other diagnostic tests.

The choice of diagnostic test depends on the aim of the diagnosis, the availability of skilled personnel, the cost, and the laboratory infrastructure [11]. Accordingly, in resource-limited settings, KK and POC-CCA are practical options, but their performance should be continually monitored due to changing disease dynamics. Previous research in Ethiopia has primarily focused on evaluating the performance of the Kato–Katz (KK) technique in moderate- to high-transmission areas, with limited attention to low-transmission settings [7, 8]. Moreover, many of these studies employed composite parasitological reference standards to assess the accuracy of KK, POC-CCA, and PCR tests, which may have resulted in less precise estimates [7–9]. Despite its high sensitivity, RT-PCR has not attained the universal validation, especially in field settings, necessary to function as a reference standard for assessing the performance of KK and POC-CCA. Therefore, given the absence of a definitive gold standard for *S. mansoni* diagnosis, the use of statistical methods such as latent class analysis (LCA) is recommended. Consequently, the present study was conducted to address the research question: “What is the diagnostic accuracy of KK, POC-CCA, and RT-PCR for detecting *S. mansoni* infections in Ethiopia, across different transmission settings, after a decade of national deworming programs?” Therefore, this study aims to assess the current field performance of these three diagnostic methods across low-, moderate-, and high-transmission settings in Ethiopia.

## Methods

### Study design, area and period

**Fig. 1:**
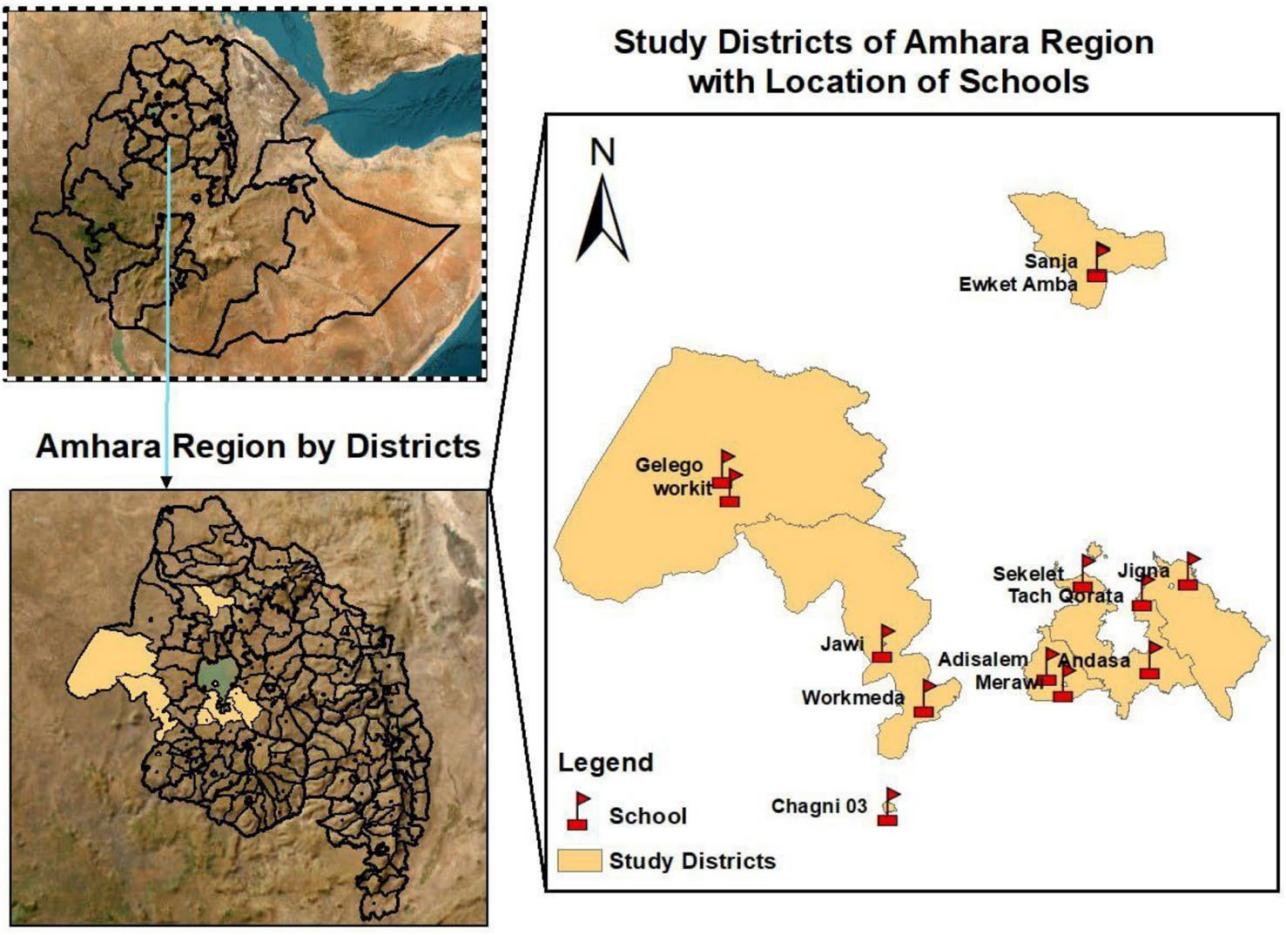
Map of the study area

From February to June 2023, a cross-sectional study was conducted in seven districts of the Amhara Regional State, northwest Ethiopia (Fig. 1). The geographic coordinates of the region are 9º–14º N and 36º–40º E. The mean annual temperature ranges from 15 ºC to 21 ºC, with an average annual rainfall of about 1165 mm [23]. According to the 2015–2020 regional mapping report, *S. mansoni* was found in 74 out of 154 mapped districts in Amhara Regional State. Seven districts had high prevalence, while moderate and low prevalence was reported in 21 districts each (Source: Amhara Regional Health Bureau).

### Study population

The source population for this study included all SACs and adults living in the districts of northwest Ethiopia. The study populations consisted of SACs attending selected primary schools during the data collection period, and adults residing in selected households.

### Eligibility criteria

Volunteer SACs (aged 6–14) and adults (aged ≥ 18) who had lived in their current residence for at least 6 months prior to data collection were eligible for inclusion. Children and adults who had taken any anthelmintic drugs within 6 months before the data collection were excluded, as were disabled individuals who were unable to respond to research questions or provide stool and/or urine samples.

### Sample size calculation

The sample size for evaluating diagnostic accuracy would have ideally been calculated based on sensitivity (or specificity) of the diagnosis tests and *S. mansoni* prevalence from previous similar studies. However, baseline data using composite results of KK, POC-CCA, and RT-PCR as reference tests are unavailable. Therefore, we used the sample size calculated using single population proportion formula for determining *S. mansoni* prevalence in the study area. After applying a design effect of 1.5 for multistage sampling, the sample size was 1270.

### Sampling technique

Districts in northwest Ethiopia were initially categorized into four groups based on the 2015–2020 SCH endemicity mapping [4]. From each of the low, moderate, and high-transmission categories, one to three districts were randomly selected. For each selected district, two primary schools were purposefully chosen due to their proximity to permanent freshwater sources. Once the schools were identified, enrollment data were collected, and the sample size was allocated proportionally across schools. A systematic sampling method was then applied using class rosters as the sampling frame. To recruit adult participants, one village near each school was purposively chosen. Household lists from health posts were used, and systematic sampling was applied to select the same number of households as the school-children recruited from the corresponding school.

### Data collection

#### Questionnaire data

Data on the sociodemographic characteristics of participants were obtained using a structured questionnaire. For children, trained health extension workers conducted face-to-face interviews at schools. In the case of adult participants, data collection was carried out either through home visits or at local health posts.

#### Stool and urine collection and examination

Participants received two labeled containers, one for stool and one for urine, after being instructed on proper sample collection. They provided approximately 5 g of stool and 3 ml of midstream urine between 9:00 AM and 12:00 AM. Urine samples were tested at the place of collection for CCA using the POC-CCA strip. Stool samples were processed the same day using the KK method at local health centers, while remaining samples were cold-shipped to the parasitology laboratory at Bahir Dar University for storage at − 80 °C and later PCR analysis. Trained laboratory technologists handled all sample collection and testing.

The urine POC-CCA and stool KK tests were performed following previously published protocols [24]. Briefly, the Schisto POC-CCA test (Rapid Medical Diagnostics, Cape Town, South Africa) was performed by adding two drops (100 µl) of urine to a horizontally placed test cassette. If CCA antigens were present, a pink test line appeared within 20 min, indicating a positive result. The presence of a pink control line confirmed test validity.

For KK smear microscopy, 2–3 g of fresh stool was passed through a 200 μm fine mesh, and the material collected was scraped off with a plastic spatula. A template with a hole, designed to hold 41.7 mg of stool, was centered on a microscope slide and filled with the sieved sample. The template was then carefully removed. A cover slip, soaked in a glycerol-malachite green solution for 24 h, was placed over the fecal material and pressed to ensure an even smear. Slides were examined for *S. mansoni* eggs, and the eggs per gram (epg) of stool were calculated by multiplying the number of eggs on the slide by 24. The epg was determined from duplicate KK slide readings, with the average taken as the final result.

#### DNA extraction and molecular detection of *S. mansoni* in stool

*Schistosoma mansoni* egg DNA was extracted from stool samples using the QIAamp Fast DNA Stool Mini kit (Qiagen, Germany) following the manufacturer’s protocol. Approximately 180–220 mg of frozen stool was mixed with 1 ml InhibitEX buffer, vortexed, and heated at 95 °C for 5 min. After centrifugation, 200 µl of the supernatant was treated with proteinase K and buffer AL, and then heated at 70 °C for 10 min. Absolute ethanol was added, and the lysate was processed through a QIAamp spin column. DNA was washed with buffers AW1 and AW2, then eluted with ATE buffer and stored at -80 °C.

DNA detection was performed using smcyt748F forward and smcyt847R reverse primers, and the smcyt785T probe targeting the cytochrome c oxidase subunit 1 (cox1) sequence in the *S. mansoni* mitochondrial genome. The primers and probe sequences, designed to amplify a 99-bp fragment of the target gene, were as follows:

- smcyt748F: 5’-CCCTGCCAAATGAAGAGAAAAC-3’
- smcyt847R: 5’-TGGGTGTGGAATTGGTTGAAC-3’
- smcyt785T: 5’-/56-FAM/CCA AAA CCA/ZEN/GAC CCC TCT CAA ATT G/3IABkFQ/-3’

DNA amplification and detection were performed using a QuantStudio 5 RT‒PCR thermocycler (Thermo Fisher Scientific, Life Technologies Holdings Pte Ltd., Singapore) and HID RT‒PCR Analysis Software v1.3. The 25 µL reaction mixture contained 12.5 µL of Hotsplit MasterMix 2X (Biotools, Madrid, Spain), 0.2 µL of 10 µM probe (Integrated DNA Technologies), 0.5 µL of 10 µM forward and reverse primers (Merck Life Science), 1.3 µL of nuclease-free water, and 10 µL of template DNA. The thermal cycling conditions were: initial hold at 95 °C for 3 min, followed by 40 cycles of 95 °C for 15 s, 60 °C for 30 s, and 72 °C for 30 s.

Positive controls, consisting of DNA from a microscopically confirmed *S. mansoni*-positive stool sample, were included in each run. A blank containing 25 µL of mastermix and a negative control with 15 µL of mastermix and 10 µL of distilled water were also included. Samples with cycle threshold (CT) values < 38 were considered positive.

#### Data quality assurance

Data collectors received training on laboratory techniques and data collection methods. A pre-test was conducted at Abiot Fana Primary School (North Mecha, Gojjam) to evaluate the questionnaire, field procedures, and familiarize the team with the tools. All laboratory procedures adhered strictly to standard operating protocols and manufacturer guidelines. Two KK smears were prepared and examined for each stool sample. Prior to use, the expiration dates of POC-CCA test kits, DNA extraction and detection kits were verified. Results from the KK and POC-CCA tests were independently read by two medical laboratory technologists, with a senior staff member resolving any discrepancies. Blank, positive, and negative controls were included in each RT-PCR run.

### Data analysis

Data entry and descriptive analysis were conducted using the Statistical Package for the Social Sciences (SPSS) version 21 (SPSS Inc., Chicago, USA). The diagnostic performance of POC-CCA and RT-PCR was evaluated using the KK method as a reference standard. Sensitivity, specificity, positive predictive value (PPV), negative predictive value (NPV), and kappa (κ) statistics were calculated using the Simple Interactive Statistical Analysis (SISA) online tool. The LCA model analysis was performed as explained elsewhere [25, 26]. In brief, a two-class LCA model was specified to estimate the diagnostic accuracy of KK, POC-CCA, and RT-PCR, using the unobserved true infection status (infected vs. non-infected) as the latent variable. The three test results were modeled as binary indicators (positive = 1, negative = 0), and conditional independence between tests was assumed given the individual’s latent (true) infection status. Parameters, including sensitivity and specificity were estimated using maximum likelihood estimation model in MPlus software version 8.10 (Statmodel). The likelihood function was constructed based on the joint probabilities of observed test result patterns; given the model parameters. To assess model fitness, we used entropy, which is calculated from the posterior probabilities of class membership for each individual; values closer to 1 indicate clearer separation between latent classes and higher classification certainty. Agreement between each diagnostic method and the reference standards was assessed using kappa statistics, interpreted according to Landis and Koch’s classification: κ ≤ 0.01 indicates no agreement; 0.01–0.20, slight; 0.21–0.40, fair; 0.41–0.60, moderate; 0.61–0.80, substantial; and 0.81–1.00, almost perfect agreement [27].

## Results

### Demographic characteristics of study participants

1192 participants provided stool and urine samples, and had complete KK, POC-CCA and RT-PCR data for analysis. Of these, 634 (53.2%) were children aged 6–14, and 558 (46.8%) were adults aged ≥ 18 years. Among the participants, 585 (49.5%) were males, while 607 (50.9%) were females. Six hundred and twelve (51.3%), 406 (34.1%) and 174 (14.6%) participants were enrolled from low-, moderate- and high-endemic districts, respectively.

### Prevalence and intensity of *S. mansoni*

**Fig. 2:**
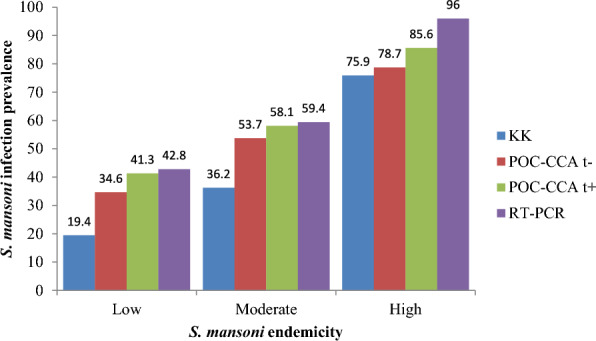
Prevalence of *S. mansoni* infection across transmission settings by different diagnostic methods

782 participants (65.6%) were positive for *S. mansoni* infection by at least one diagnostic test when POC-CCA trace results were interpreted as positive (t +) and 757 participants (63.5%) when interpreted as negative (t−). The prevalence of infection varied across different diagnostic methods: 33.4% by KK, 53.5% by POC-CCA t+, 47.6% by POC-CCA t-, and 56.2% by RT-PCR. The prevalence rates also differed according to the transmission endemicity (Fig. 2).

**Fig. 3:**
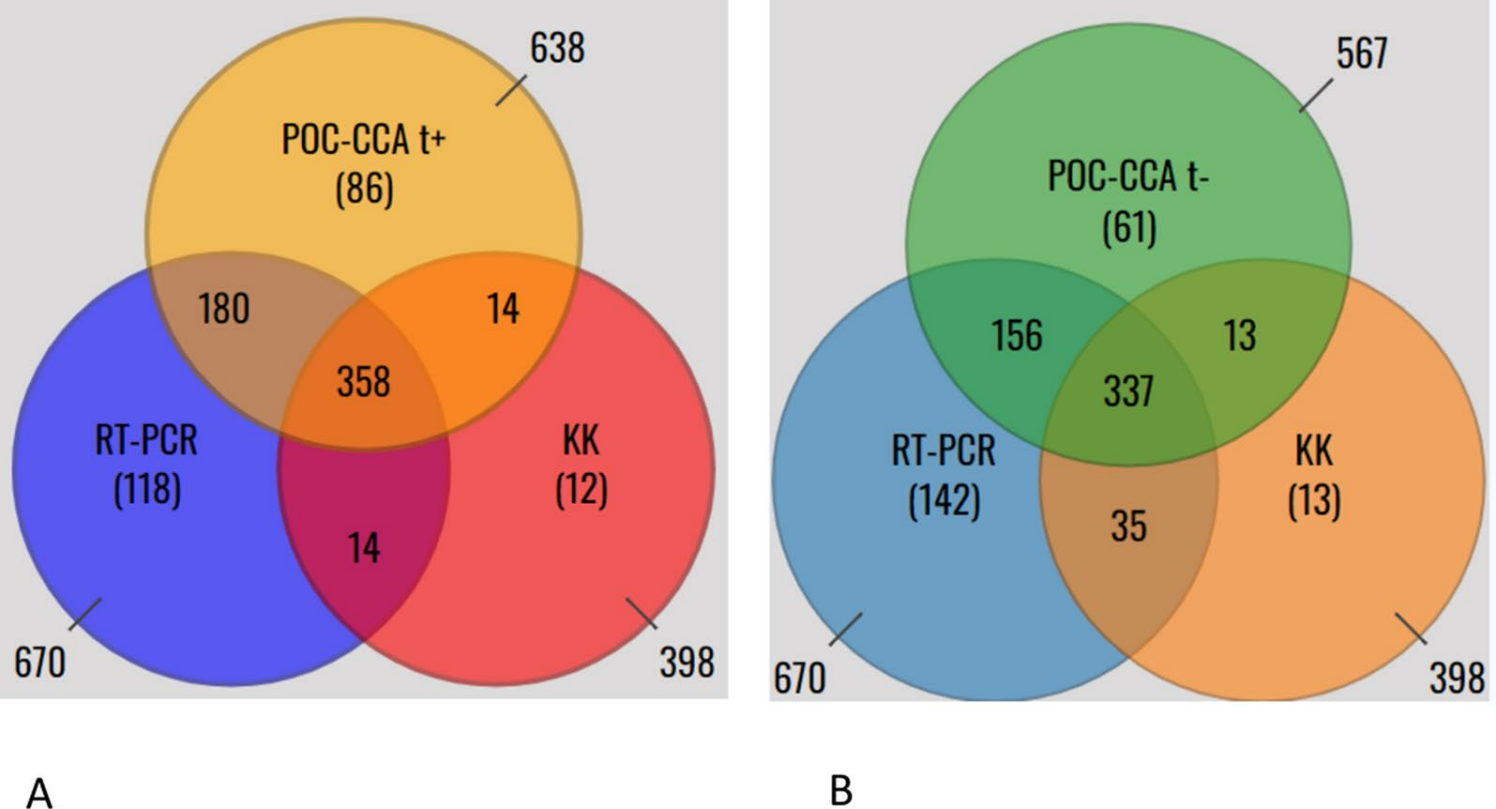
Venn diagram showing the relationship in detecting *S. mansoni* by the three diagnostic tests when POC-CCA t+ (**A**) and t− (**B**)

When POC-CCA trace was considered positive, of the 782 participants who tested positive, 358 (45.8%) were positive by all three diagnostic tests. In addition, 118 (15.1%) were positive only by RT-PCR, 86 (11.0%) by POC-CCA, and 12 (1.5%) by KK alone. When POC-CCA trace results were interpreted as negative, 44.5% of the positive participants were positive by all three diagnostic tests (Fig. 3).

A high level of discrepancy was observed between the results of KK and POC-CCA. Specifically, 217 samples that were reactive by POC-CCA tested negative by KK, while 26 egg-positive samples were non-reactive for CCA. Similarly, RT-PCR yielded positive in 298 egg-negative samples (Table 1).

**Table 1:** Discrepancies in results between diagnostic tests

	POC-CCA result			Total
	R	T	NR	
KK				
Positive	350	22	26	398
Negative	217	49	528	794
Total	567	71	554	1192
RT-PCR				
Positive	493	45	132	670
Negative	74	26	422	522
Total	567	71	554	1192

	RT-PCR			
	Positive	Negative	Total	
KK				
Positive	372	26	398	
Negative	298	496	794	
Total	670	522	1192	

**Fig. 4:**
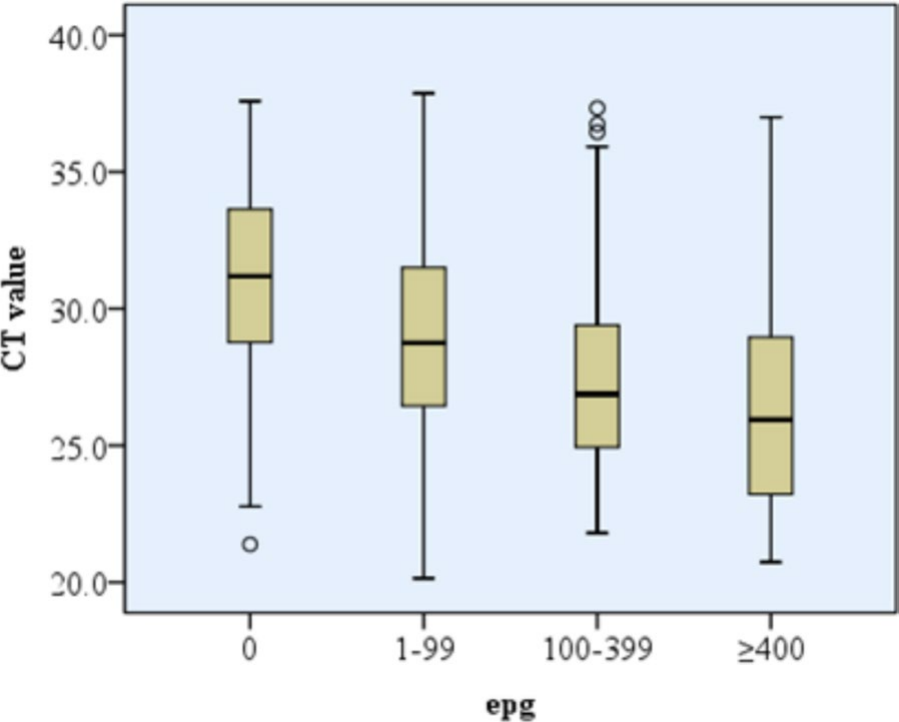
Correlation between CT values of RT-PCR with epg of KK

Out of 398 individuals who tested positive for *S. mansoni* using duplicate KK smears, 266 (66.8%) had light-intensity infections, 100 (25.1%) had moderate intensity, and 32 (8.1%) had heavy-intensity infections, with an average egg count of 130.3 epg ± 154.9. In urine-based POC-CCA testing, 567 participants (47.6%) had a clearly visible (reactive) test band, 71 (6.0%) showed a faint (trace) band, and 554 (46.4%) had no visible band (non-reactive) (Table 2). Among 670 individuals who tested positive via RT-PCR, 295 (44.0%) had light infections (CT > 30) and 375 (56.0%) had heavy infections (CT ≤ 30). When CT values of RT-PCR were compared with epg of KK, there was a significant negative correlation between them (spearman’s correlation coefficient = − 0.402, *p* < 0.001) (Fig. 4).

**Table 2:** Performance of KK, POC-CCA and RT-PCR at varying transmission settings

Index test	Endemicity	Sensitivity % (95% CI)	Specificity % (95% CI)	PPV % (95% CI)	NPV % (95% CI)	Kappa value (95% CI)
Latent class analysis						
KK	Low	54.6(45.9–63.3)	97.5(95.6–99.5)	91.8(85.6–98.0)	81.0(76.6–85.4)	0.58(0.49–0.68)
	Moderate	67.0(58.9–75.1)	96.4(93.0–99.7)	95.3( 91–99.6)	72.8(65.8–79.7)	0.63(0.51–0.74)
	High	88.6(82.1–95.1)	100	100	59.5(40.6–78.5)	0.69(0.51–0.87)
	Total	69.4(64.6–74.3)	97.1(95.5–98.8)	95.6(93.1–98.1)	77.8(74.1–81.6)	0.67(0.61–0.74)
POC-CCA	Low	97.6(94.9–100)	86.0(81.7–90.3)	77.8(71.3–84.3)	98.6(97.0–100)	0.79(0.69–0.89)
	Moderate	93.4(89.1–97.7)	78.9( 71.5–86.2)	82.8(76.7–88.9)	91.6(86.3–97.0)	0.73(0.60–0.85)
	High	100	100	100	100	1.0(0.81–1.0)
	Total	97.2(95.4–98.9)	83.4(79.7–87.1)	84.1(80.5–87.7)	97.0(95.2–98.9)	0.80(0.73–0.87)
RT-PCR	Low	93.7(89.4–97.9)	84.2(79.9–88.4)	72.2(65.3–79.0)	96.8(94.6–99.0)	0.72(0.62–0.81)
	Moderate	96.2(93.0–99.5)	79.4(72.1–86.6)	83.6(77.7–89.5)	95.1(90.8–99.3)	0.76(0.64–0.88)
	High	100	28.0(5.6–50.4)	89.2(83.2–95.2)	100	0.4(0.25–0.55)
	Total	97.2(95.4–98.9)	78.3(74.2–82.4)	80.2(76.4–84.0)	96.8(94.9–98.8)	0.75(0.68–0.82)
Kato–Katz as a gold standard						
POC-CCA	Low	90.8(84.1–97.4)	66.7(61.0–72.3)	42.7(34.9–50.5)	96.3(93.6–99.0)	0.41(0.32–0.50)
	Moderate	89.8(83.6–96.0)	59.9(52.2–67.5)	55.9(47.9–64.0)	91.2(85.7–96.6)	0.44(0.33–0.55)
	High	100	59.5(40.6–78.5)	88.6(82.1–95.1)	100	0.69(0.51–0.87)
	Total	93.5(90.4–96.6)	66.5(62.3–70.7)	58.3(53.4–63.2)	95.3(93.1–97.6)	0.52(0.46–0.56)
RT-PCR	Low	90.5(83.7–97.3)	68(62.7–73.2)	39.9(32.4–47.5)	96.8(94.5–99.2)	0.39(0.31–0.48)
	Moderate	92.5(87.1–97.9)	59.5(51.8–67.1)	56.4(48.4–64.4)	93.3(88.5–98.2)	0.46(0.35–0.57)
	High	100	16.7(2.3–31.0)	79.0(71.2–86.9)	100	0.23(0.11–0.35)
	Total	93.5(90.4–96.6)	62.5(58.2–66.8)	55.5(50.7–60.3)	95(92.6–97.4)	0.48(0.41–0.54)

### Performance of diagnostic tests

The POC-CCA test demonstrated a high sensitivity of 93.5% (95% CI: 90.4–96.6), with slight variations depending on the level of disease transmission: 90.8% in low, 89.8% in moderate, and 100% in high-endemic areas, when compared to the KK reference method. However, its specificity was relatively low at 66.5% (95% CI: 62.3–70.7), as was its PPV, which stood at 58.3% (95% CI: 53.4–63.2). The test showed moderate agreement with KK, with a kappa statistic of 0.52. Similarly, RT-PCR showed comparable performance, with the same sensitivity of 93.5% (95% CI: 90.4–96.6) and a slightly lower specificity of 62.5% (95% CI: 58.2–66.8) when assessed against the KK reference. When evaluated using LCA reference, both the POC-CCA and RT-PCR tests showed equally high sensitivity at 97.2% (95% CI: 95.4–98.9), representing a 4.3% increase compared to their sensitivity based on the KK reference. In contrast, the KK method exhibited much lower sensitivity at 69.4% (95% CI: 64.6–74.3), with significant variation depending endemicity—54.6% in low, 67.0% in moderate, and 88.6% in high-endemic areas. However, KK maintained high specificity, consistently exceeding 95% across all transmission settings (Table 2).

## Discussion

The Amhara Regional State is considered to have moderate endemicity for *S. mansoni*, with pooled prevalence estimates from previous studies using parasitological diagnostics ranging between 17.5% and 41.1% [28–31]. In the present study, the prevalence detected using the KK method was similarly moderate (33.4%), but there was considerable variation across transmission areas, ranging from 19.4% in low-endemic to 75.9% in high-endemic areas. This variation is due to the focal nature of the parasite’s distribution, influenced by factors such as the availability of suitable water bodies, water flow, intermediate snail hosts, and environmental, climatic, and physicochemical conditions [32]. When combining results from the KK, POC-CCA, and RT-PCR tests, the prevalence was nearly double, with 65.6% positive for POC-CCA t+ and 63.5% for POC-CCA t-. Both POC-CCA and RT-PCR are known to be more sensitive than the KK method [6, 20, 33], and their use significantly increased the prevalence as supported by previous studies in Africa [22, 32]. In line with this, reviews of previous studies revealed that the urine POC-CCA prevalence reached three–eightfold higher than that of KK based on the *S. mansoni* endemicity of data collection areas [35, 36].

The KK diagnostic method demonstrates significantly lower sensitivity in low-transmission settings compared to high-transmission areas, raising concerns about its continued use in the post-intervention era. In low-transmission areas, KK failed to detect 60.3% of RT-PCR positive cases and 57.3% of POC-CCA positives, whereas in high-transmission areas, the false-negative rates were substantially lower at 21.0% and 11.4%, respectively (Supplementary file S1). This discrepancy is reflected in the sensitivity of KK, which increases from 54.6% in low-transmission areas to just 88.6% in high-transmission contexts. Overall, KK showed a sensitivity of 69.4% against the LCA reference standard, markedly lower than both that of RT-PCR and POC-CCA, which each achieved 97.2% (Table 2). These findings underscore the limitations of KK in detecting low-intensity infections and suggest that more sensitive diagnostic tools such as RT-PCR and POC-CCA are better suited for surveillance and monitoring in areas approaching elimination.

The low sensitivity of KK can be attributed to factors such as the heterogeneous distribution of eggs within the stool sample, variability in day-to-day egg excretion, anti-fecundity immunity as well as the possibility of single-sex or pre-patent infections [10–12, 37, 38]. The KK sensitivity is closely related to the egg load or infection intensity, with its sensitivity being lowest in low-transmission areas compared to moderate and high-transmission areas, which was evident in this study (54.6% in low, 67.0% in moderate and 88.6% in high-transmission settings). Despite RT-PCR is more sensitive than KK, it failed to amplify DNA in 26 samples that were egg-positive in this study, which was in line with a previous study where nine out of 215 egg-positive samples were negative by PCR [19]. This discrepancy could be due to intra-sample variability in egg distribution (eggs might be present in the part of the stool used for KK but absent in the part used for PCR from the same sample), the presence of high background DNA from other biological materials in the stool, or genetic variation of *S. mansoni* might affect the attachment of primers and the probe to the template DNA [34, 39].

On the other hand, the specificity of RT-PCR tends to decline as endemicity increases, and this may be attributed to several factors. First, in highly endemic areas, individuals are often repeatedly exposed to schistosoma infection, leading to partial immunity and reduced worm burdens. These light infections may not be detected by conventional reference methods such as the KK, resulting in a higher rate of false-positive RT-PCR findings and, consequently, lower specificity. Second, RT-PCR may detect cell-free egg-derived DNA in stool samples from individuals who are no longer actively excreting viable eggs or CCA. This situation can arise in individuals who have been cured, but still harbor trapped eggs within intestinal granulomas. Over time, these eggs may undergo disintegration, releasing DNA into the intestinal lumen, which is then picked up by RT-PCR assays despite the absence of active infection [20, 40, 41].

The POC-CCA t+ test turned positive results in 217 out of 794 egg-negative samples. It demonstrated higher sensitivity than KK (97.2% vs. 69.4%) against LCA reference, which is in line with previous findings [14, 26, 36, 42]. This improved performance is largely because POC-CCA test overcomes several limitations of the KK technique. Unlike KK, which requires mixed-sex infections to detect eggs, POC-CCA can detect CCA even in single-sex (male or female) worm infections. In addition, both juvenile and adult worms release CCA, whereas KK only becomes positive after the worms mature and begin laying eggs [36, 43]. The POC-CCA test is also capable of detecting much lower antigen concentrations than the egg threshold required for KK detection, and it exhibits less daily variability in CCA excretion compared to the fluctuations in egg output, making its results more consistent [15, 16, 34, 44, 45]. However, the higher rate of positive results by POC-CCA also includes a considerable number of false positives. This can be attributed to cross-reactivity with other helminth infections, use of diuretics, urinary tract infections, pregnancy and the presence of blood in urine [10–12, 46, 47]. Supporting this, the POC-CCA had a lower (than that of KK) specificity of 83.4% and 69.4% against LCA and KK references, respectively. Conversely, among 398 samples that were egg-positive, 26 (6.5%) were negative by POC-CCA. Notably, 23 of these had light infection intensities (epg < 100), reinforcing findings from earlier studies that POC-CCA has reduced sensitivity in low-transmission areas and in cases of light infections [10, 48, 49]. In contrary to this, in the present study, there was only little difference in POC-CCA sensitivity across transmission settings (Table 2).

The commercially available POC-CCA is recommended for routine use in Ethiopia because it had good sensitivity and acceptable specificity, which makes it by far more accurate than the currently in-use KK. It is also user friendly and urine-based that collection of urine is easier than stool and more acceptable by the community thereby increasing the compliance during sample collection. Worrell and colleagues estimated the cost POC-CCA test considering supply, capital, labor, transport and overhead costs and found it $ 7.26 per test, which was nearly equal to the cost of a single KK test $ 6.89. The cost per test becomes lower compared to the recommended duplicate KK per sample [50].

However, the interpretation of weakly reactive (trace) results in the POC-CCA test remains a debated issue. As trace results are neither clearly positive nor negative, they can significantly affect prevalence estimates and control decisions. In this study, treating trace results as positive versus negative resulted in a 5.9% difference in prevalence, highlighting the need for standardized interpretation—especially when guiding MDA. The fact that 21 out of 71 POC-CCA trace samples were positive by both KK and RT-PCR tests while 25 were negative by both tests suggest it is difficult to conclude about the interpretation of each trace result as supported by a previous study [34]. Hence, the existing literature offers differing perspectives based on epidemiological context. In high-transmission settings, like Ethiopia, treating trace results as positive increases sensitivity and ensures treatment for individuals with low-intensity infections often missed by the KK method [51]. This is supported by the present study, where 45 of the 71 POC-CCA trace results (31 with CT > 30) were positive by RT-PCR, and 22 (18 with epg < 100) were positive by KK. However, in low-endemic or elimination settings, considering trace results as negative may prevent unnecessary treatment and resource waste [37]. Among the 41 POC-CCA trace samples from low-transmission areas, 34 were egg-negative and 19 were RT-PCR-negative, suggesting that trace results are more likely non-infected in such settings. To resolve this, researchers have developed a user-friendly scoring method called G-scores to provide a standardized, quantitative interpretation of the POC-CCA test. This system uses 10 POC-CCA cassettes with inkjet-printed strips showing varying test line intensities, allowing results to be graded on a scale from G1 to G10 [52]. However, this reference color scale is not included in the commercial test kits and is only available to researchers upon request. Interpretation strategies should therefore align with local epidemiology and program goals to optimize the impact of the POC-CCA test.

Unlike results of a previous study which showed that POC-CCA is more sensitive than RT-PCR [34], the two tests have comparable sensitivity in the present study (Table 2). Variations in the duration (pre-patent vs patent) and intensity of infection among study participants might bring the difference. If there are significant number of participants with pre-patent or light-intensity infections, the POC-CCA will be more sensitive than POC-CCA [34]. As shown in Fig. 2, RT-PCR is highly dependent on the intensity of infection and the present study showed a significant inverse correlation of CT value with epg (*r* = − 0.402, *p* < 0.001) which goes in line with previous study (*r* = − 0.66, *p* < 0.001) [19]. In general, despite none of the three tests are perfectly accurate, the POC-CCA is by far a more accurate point-of-care test than KK that can be practiced in field settings.

This study provides critical insights into the diagnostic performance of KK, POC-CCA and RT-PCR for the detection of *S. mansoni* in varying transmission settings within the Amhara Region of Ethiopia. By incorporating LCA as a reference, the study offers a more accurate assessment of test sensitivity and specificity in the absence of a true gold standard. Furthermore, it highlights the limitations of KK in low-endemic settings, the consistent sensitivity but reduced specificity of POC-CCA, and the high diagnostic accuracy but limited field applicability of RT-PCR. The stratified sampling approach across low, moderate, and high-transmission districts adds robustness to the findings and ensures generalizability across similar endemic regions. Ultimately, the study provides evidence-based recommendations supporting the integration of the POC-CCA test into Ethiopia’s schistosomiasis control and elimination strategies, contributing to improved disease surveillance, diagnosis, and programmatic decision-making. However, the present study is not without limitations. We did not assess day-to-day variations in CCA excretion, nor did we apply a scoring system to interpret POC-CCA results.

## Conclusion

The findings of this study highlight that KK has low sensitivity and undermines accurate prevalence estimation, especially in low-transmission areas. In contrast, the POC-CCA test demonstrates consistently higher sensitivity across all transmission settings, making it a more reliable option for case detection and large-scale screening. Therefore, we recommend the adoption of the POC-CCA test in Ethiopia’s national SCH control and elimination programs. However, to improve the consistency and utility of this test, especially in the interpretation of trace results, the implementation of a standardized scoring system—such as the G-scores—is strongly advised. The RT-PCR offers high diagnostic accuracy. However, because of its technical demands and resource intensity, its use should be reserved for confirmatory testing and epidemiological surveillance in specialized settings rather than for routine diagnosis. A diagnostic approach tailored to the local epidemiological context, combining POC-CCA for broad screening with RT-PCR for targeted validation, would enhance the effectiveness of SCH control strategies in Ethiopia and other resource-limited endemic countries.

## Supplementary Information

Supplementary file 1.

## Data Availability

All data are available from the corresponding author. No datasets were generated or analyzed during the current study.

## Abbreviations

CCA: Circulating cathodic antigen
epg: Eggs per gram of stool
CT: Cycle threshold
KK: Kato–Katz
NPV: Negative predictive value
POC-CCA: Point of care circulating cathodic antigen
PPV: Positive predictive value
RT-PCR: Real-time polymerase chain reaction
SCH: Schistosomiasis
WHO: World Health Organization
