# Supplementary material for: Comparative performance of Kato–Katz, POC-CCA and real-time PCR in detecting Schistosoma mansoni infection at different endemicity settings in northwest Ethiopia: a cross-sectional study
Source: Trop Med Health. 2025 Aug 4;53:103. doi: 10.1186/s41182-025-00777-7 (PMC12323140; doi:10.1186/s41182-025-00777-7)
Supplement: Supplementary file 1 [file 41182_2025_777_MOESM1_ESM.docx]

**1. Overall comparison of diagnostic tests**

|  | | **Latent class** |  |
| --- | --- | --- | --- |
|  |  | Infected | Not infected |
| **KK** | Positive | 393 | 18 |
|  | Negative | 173 | 608 |
|  | | **KK** | |
|  |  | Positive | Negative |
| **POC-CCA** | Positive | 372 | 266 |
|  | Negative | 26 | 528 |
|  | | **Latent class** | |
|  |  | Infected | Not infected |
| **POC-CCA** | positive | 495 | 75 |
|  | negative | 46 | 576 |
|  | | **KK** | |
|  |  | Positive | Negative |
| **PCR** | Positive | 372 | 298 |
|  | Negative | 26 | 496 |
|  | | **Latent class** | |
|  |  | Infected | Not infected |
| **RT-PCR** | Positive | 525 | 149 |
|  | Negative | 16 | 502 |

**2. Comparison of diagnostic tests in low transmission settings**

|  | | | **Latent class** | |
| --- | --- | --- | --- | --- |
|  |  |  | Infected | Not infected |
| **KK** | Positive | | 112 | 10 |
|  | Negative | | 93 | 397 |
|  | | | **KK** | |
|  |  |  | Positive | Negative |
| **POC-CCA** | Positive | | 108 | 145 |
|  | Negative | | 11 | 348 |
|  | | | **Latent class** | |
|  |  |  | Infected | Not infected |
| **POC-CCA** | Positive | | 200 | 57 |
|  | Negative | | 5 | 350 |
|  | | | **KK** | |
|  |  |  | Positive | Negative |
| **PCR** | | Positive | 105 | 158 |
|  |  | Negative | 11 | 335 |
|  | | | **Latent class** | |
|  |  |  | Infected | Not infected |
| **RT-PCR** | | Positive | 192 | 74 |
|  |  | Negative | 13 | 333 |

**3. Comparison of diagnostic tests in moderate transmission settings**

|  | | **Latent class** | |
| --- | --- | --- | --- |
|  |  | Infected | Not infected |
| **KK** | Positive | 142 | 7 |
|  | Negative | 70 | 187 |
|  | | **KK** | |
|  |  | Positive | Negative |
| **POC-CCA** | Positive | 132 | 104 |
|  | Negative | 15 | 155 |
|  | | **PCR** | |
|  |  | Positive | Negative |
|  | | **Latent class** | |
|  |  | Infected | Not infected |
| **POC-CCA** | Positive | 198 | 41 |
|  | Negative | 14 | 153 |
|  | | **KK** | |
|  |  | Positive | Negative |
| **PCR** | Positive | 136 | 105 |
|  | Negative | 11 | 154 |
|  | | **Latent class** | |
|  |  | Infected | Not infected |
| **RT-PCR** | Positive | 204 | 40 |
|  | Negative | 8 | 154 |

**4. Comparison of diagnostic tests in high transmission settings**

|  | | **Latent class** | |
| --- | --- | --- | --- |
|  |  | Infected | Not infected |
| **KK** | Positive | 132 | 0 |
|  | Negative | 17 | 25 |
|  | | **KK** | |
|  |  | Positive | Negative |
| **POC-CCA** | Positive | 132 | 17 |
|  | Negative | 0 | 25 |
|  | | **Latent class** | |
|  |  | Infected | Not infected |
| **POC-CCA** | Positive | 149 | 0 |
|  | Negative | 0 | 25 |
|  | | **KK** | |
|  |  | Positive | Negative |
| **PCR** | Positive | 132 | 35 |
|  | Negative | 0 | 7 |
|  | | **Latent class** | |
|  |  | Infected | Not infected |
| **RT-PCR** | Positive | 149 | 18 |
|  | Negative | 0 | 7 |
